# Gate control of sensory neurotransmission in peripheral ganglia by proprioceptive sensory neurons

**DOI:** 10.1093/brain/awad182

**Published:** 2023-05-30

**Authors:** Alice M Fuller, Ana Luiz, Naxi Tian, Manuel Arcangeletti, Federico Iseppon, Jane E Sexton, Queensta Millet, Sara Caxaria, Niloofar Ketabi, Petek Celik, John N Wood, Shafaq Sikandar

**Affiliations:** William Harvey Research Institute, Queen Mary University of London, London EC1M 6BQ, UK; Wolfson Institute for Biomedical Research, University College London, London WC1E 6BT, UK; Wolfson Institute for Biomedical Research, University College London, London WC1E 6BT, UK; Wolfson Institute for Biomedical Research, University College London, London WC1E 6BT, UK; Wolfson Institute for Biomedical Research, University College London, London WC1E 6BT, UK; Wolfson Institute for Biomedical Research, University College London, London WC1E 6BT, UK; Wolfson Institute for Biomedical Research, University College London, London WC1E 6BT, UK; Wolfson Institute for Biomedical Research, University College London, London WC1E 6BT, UK; William Harvey Research Institute, Queen Mary University of London, London EC1M 6BQ, UK; William Harvey Research Institute, Queen Mary University of London, London EC1M 6BQ, UK; William Harvey Research Institute, Queen Mary University of London, London EC1M 6BQ, UK; Wolfson Institute for Biomedical Research, University College London, London WC1E 6BT, UK; William Harvey Research Institute, Queen Mary University of London, London EC1M 6BQ, UK

**Keywords:** parvalbumin, gate control, nociception, gamma-aminobutyric acid, dorsal root ganglia

## Abstract

Melzak and Wall’s gate control theory proposed that innocuous input into the dorsal horn of the spinal cord represses pain-inducing nociceptive input. Here we show that input from proprioceptive parvalbumin-expressing sensory neurons tonically represses nociceptor activation within dorsal root ganglia. Deletion of parvalbumin-positive sensory neurons leads to enhanced nociceptor activity measured with GCaMP3, increased input into wide dynamic range neurons of the spinal cord and increased acute and spontaneous pain behaviour, as well as potentiated innocuous sensation. Parvalbumin-positive sensory neurons express the enzymes and transporters necessary to produce vesicular GABA that is known to be released from depolarized somata. These observations support the view that gate control mechanisms occur peripherally within dorsal root ganglia.

## Teaser

Fuller *et al.* describe a novel role of proprioceptive parvalbumin-expressing sensory neurons in the tonic inhibition of nociceptive signalling within dorsal root ganglia, and use *in vivo* calcium imaging, electrophysiology and pain behaviour to demonstrate the existence of a peripheral pain gate.

## Introduction

Gassar’s mid-20th century observation that pain could be relieved through activation of rapidly conducting primary afferent fibres^1^ was translated into a ‘new theory of pain’ by Melzack and Wall^2^ in 1965. They suggested that within the spinal cord, inhibitory cells control transmission of nociceptive information to higher centres through their activation by innocuous primary afferent neurons. There is a consensus view that the underlying principle of gate control of nociceptive input by non-nociceptive sensory neurons is correct.^3^ Previous studies also demonstrate the functional expression of GABA receptors in primary afferent neurons, supporting the framework for an inhibitory circuit on spinal cord excitability mediated by dorsal root ganglia neurons.^4,5^ Recent work from Gamper and colleagues showed inhibitory neurotransmitter GABA is released from sensory neurons within dorsal root ganglia when they are depolarized.^6^ Release from vesicular pools was evoked by high potassium as well as pro-nociceptive stimuli as varied as ATP, bradykinin and capsaicin. GABA receptors were also found to be expressed by sensory neuron somata, and exogenous GABA applied within dorsal root ganglia (DRG) diminishes nociceptive input and pain behaviour in mice, suggesting that gate control may occur within dorsal root ganglia.^6^

Advillin is uniquely expressed within peripheral sensory neurons^7^ and parvalbumin is a calcium binding protein that is an established marker of NT-3-dependent proprioceptive neurons.^8^ We used the advillin promoter to drive diphtheria toxin downstream of a floxed stop signal, which results in the expression of the toxin and the death of parvalbumin-positive sensory neurons whilst CNS parvalbumin-expressing neurons remain unaffected (Supplementary Fig. 1). Using this loss-of-function approach we demonstrate an inhibitory drive mediated by parvalbumin-expressing sensory neurons on neuronal excitability at the level of the soma, dorsal horn and on behavioural outcomes *in vivo*.

## Materials and methods

### Animals

All animal procedures were performed by licensed individuals and conformed to UK Home Office regulations in accordance with the Animals (Scientific Procedures) Act 1986. Parvalbumin-Cre (PV-Cre) mice (Jackson Laboratory stock no. 017320) were crossed to Advillin-loxP-tdTomato-Stop-loxP-DTA mice^7^ to generate progeny termed PV^DTA^. Progeny were bred to Pirt-GCaMP3-expressing mice^9,10^ for GCaMP imaging experiments. Test mice used for *in vivo* imaging experiments were PV^DTA^; GCaMP3. All experiments were performed on adult mice (male and female) heterozygous for each genotype, where applicable.

### Immunohistochemistry

Littermate and PV^DTA^ mice were transcardially perfused with 4% paraformaldehyde. Isolated DRG were post-fixed at 4°C for 2 h, followed by 30% w/v sucrose in 1× PBS at 4°C overnight before being mounted in O.C.T. medium (Tissue-Tek) and stored at −80°C. Sections (10 µm) were washed in PBST followed by incubation with blocking buffer (10% normal goat serum) for 60 min at room temperature. Tissue was then incubated with anti-parvalbumin antibody (Abcam, ab11427) diluted in blocking buffer (1:700), at 4°C overnight. Tissue was incubated with an Alexa Fluor 488 goat anti-rabbit secondary antibody (Invitrogen, A11008) diluted in blocking buffer (1:1000) for 2 h at room temperature. Images were captured from Vectashield® mounted slides using an upright Leica SP8 confocal microscope (Leica) using laser lines 488 (PV:Alexa Fluor 488), and 552 nm (tdTomato). LASX software (Leica) was used for cell counts.

### Quantitative RT-PCR

cDNA was synthesized from ∼1 μg of isolated RNA, using the iScript™ reverse transcription supermix for RT-qPCR kit (Bio-Rad). To detect relative expression levels of genes in the DRG, qPCR reactions were set up using SYBR Green Superimx (Bio-Rad) according to manufacturer’s instructions, and using the following primer pairs: *GAPDH* fwd: TGCGACTTCAACAGCAACTC, *GAPDH* rev: CTTGCTCAGTGTCCTTGCTG; *Pvalb* fwd: CCCGCTCAAACAGTTGCAGG, *Pvalb* rev: TCAGAATGGACCCCAGCTCAT. The 2^−ΔΔCt^ method was used to analyse the threshold cycle (Ct) values obtained for the *Pvalb* primer pairs normalized to the values obtained from *GAPDH* primer pairs, to give a relative expression level.

### Behavioural studies

Standardized pain behaviour assays were used as previously described by the group.^11^ Further details for behavioural tests assessing motor coordination and sensory function are described in the Supplementary material, ‘Methods’ section.

### *In vivo* GCaMP imaging

Briefly, a dorsal laminectomy was performed in anaesthetized mice (120 mg/kg ketamine) (Fort Dodge Animals Health Ltd.) and 1.2 mg/kg medetomidine (Orion Pharma) to expose L4 DRG.^12^ The area was continuously perfused with artificial CSF containing: 120 mM NaCl, 3 mM KCl, 1.1 mM CaCl_2_, 10 mM glucose, 0.6 mM NaH_2_PO_4_, 0.8 mM MgSO_4_, 18 mM NaHCO_3_. An upright Leica SP8 confocal microscope was used for imaging GCaMP excitation at 488 nm (<5% laser power). All images were acquired at a bidirectional scan speed of 800 Hz, with a frame rate of 1.54 frames/s and a resolution of 512 × 512 pixels, and activity in response to ipsilateral hind paw stimulation was recorded. Details on stimulus application and image analysis are described in the Supplementary material, ‘Methods’ section.

### *In vivo* electrophysiology

A laminectomy was performed in anaesthetized mice (1.5–1.7% isoflurane in 0.5 l/min N_2_O and 1.5 l/min O_2_) to expose the L3–5 region of the spinal cord. Wide dynamic range (WDR) neurons in the deep dorsal horn (lamina III–V 200–600 μm depth) were selected for recording using a Parylene-coated tungsten electrode (A-M Systems Inc.). Neuronal activity was visualized using an oscilloscope. The sampling rate was 20 000 Hz, and data were analysed on a spike amplitude and waveform basis using a CED 1401 interface coupled to Spike2 software (Cambridge Electronic Design Ltd.). Further details on dorsal horn neuronal recordings are provided in the Supplementary material, ‘Methods’ section.

### Statistical analysis

Statistical analysis was performed where appropriate using GraphPad Prism 9 Software. The mean ± standard error of the mean (SEM) was calculated for all data where applicable. To compare groups Student’s unpaired *t*-tests, Student’s paired *t*-tests, regular two-way ANOVA with Tukey’s multiple comparison test, and regular one-way ANOVA with Dunnet’s multiple comparison test, were performed. Mann-Whitney test was used for comparisons within discrete datasets. Data are presented as mean ± SEM. *P < 0.05; **P < 0.01; ***P < 0.001; ****P < 0.0001.

## Results

### Increased peripheral and central neuronal excitability in PV^DTA^ mice

**Figure 1:**
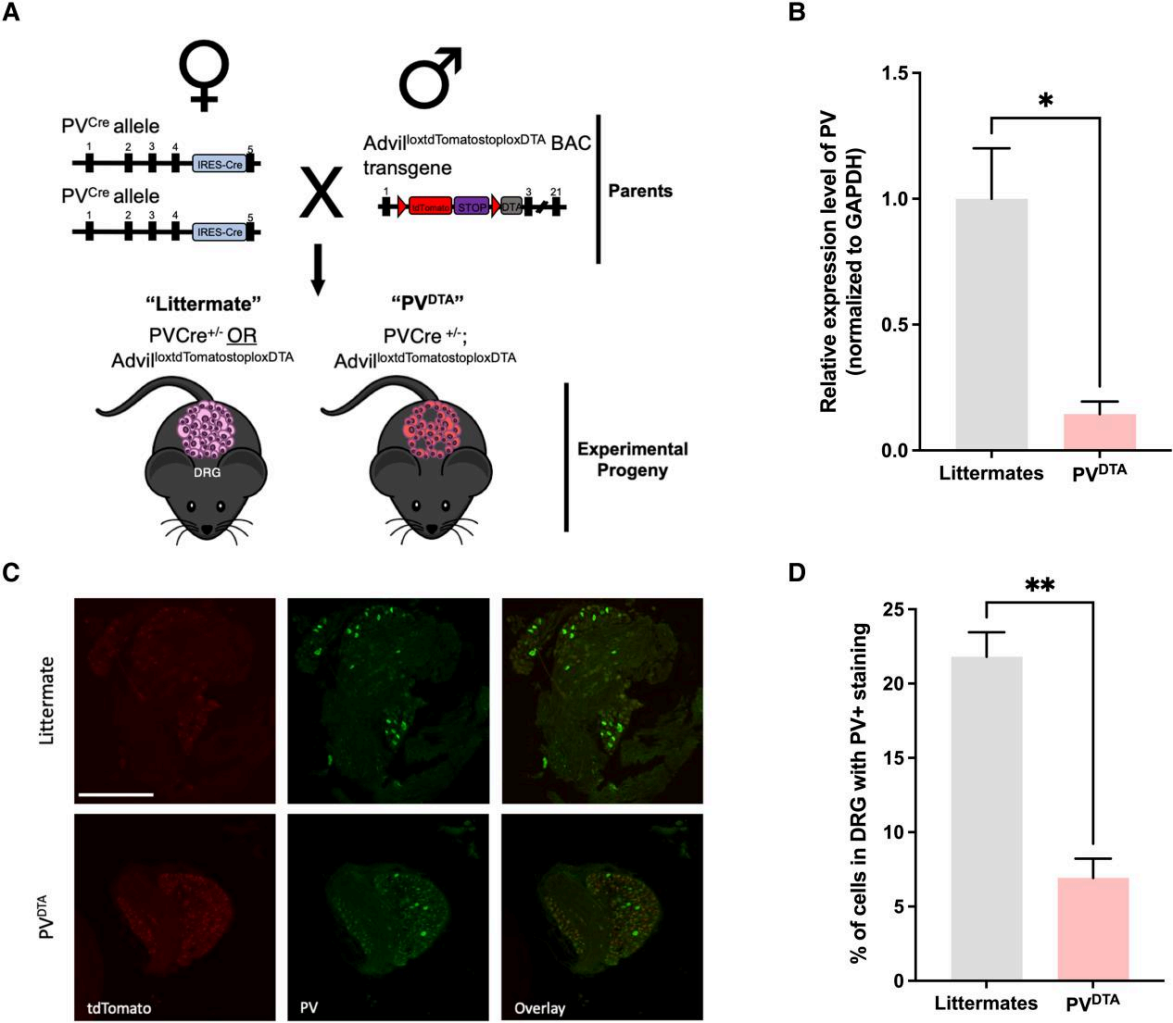
**Loss of parvalbumin (PV) mRNA and protein in the DRG of PV^DTA^ mice**. (**A**) Breeding strategy for PV^DTA^ mice. The red arrowheads denote loxP sites. The dorsal root ganglion (DRG) neurons of PV^DTA^ progeny express the red fluorescent reporter tdTomato in PV−, Advil+ neurons. (**B**) qRT-PCR showing PV mRNA expression in the DRG of PV^DTA^ mice is significantly reduced compared to littermate controls, relative to *GAPDH* expression. (**C**) Representative DRG tissue sections stained with anti-PV antibody. TdTomato represents the advillin-expressing neuronal population. Scale bar = 500 µm. (**D**) Quantification of PV+ neurons in the DRG of littermate and PV^DTA^ animals, expressed as a percentage of the total number of DRG neurons (*n* = 3 per group). Data are mean ± SEM with unpaired Student’s *t*-test. *P < 0.05; **P < 0.01; ***P < 0.001; ****P < 0.0001.

**Figure 2:**
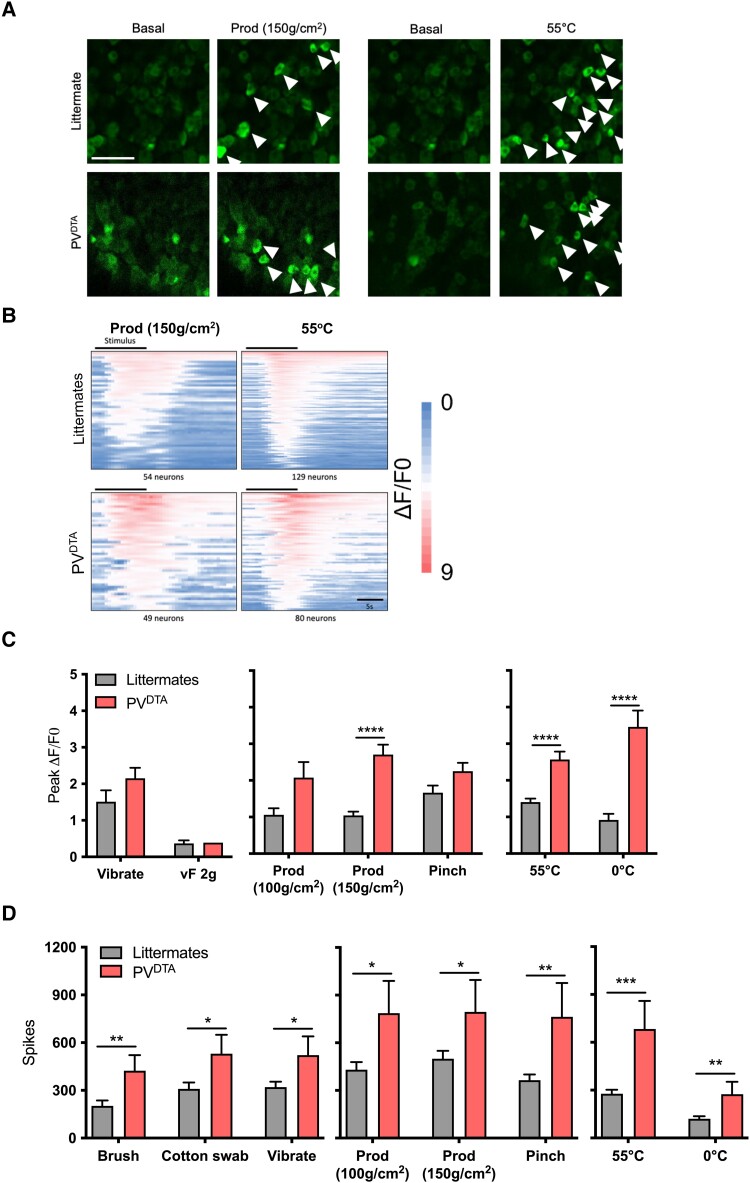
**L4 DRG neurons show enhanced activity with noxious peripheral stimulation in PV^DTA^ mice, as do wide dynamic range neurons in the dorsal horn**. (**A**) Representative images of *in vivo* calcium imaging of DRG neurons expressing GCaMP3, and their response to 150 g/cm^2^ prod or 55°C applied to ipsilateral hind paw. Scale bar = 100 µm. (**B**) Heat map demonstrating responses to mechanical prod and 55°C heat stimulation. (**C**) Peak fluorescence of DRG neurons in littermate (*n* = 263 cells) and PV^DTA^ mice (*n* = 179 cells). The latter exhibit increased calcium transients evoked by 150 g/cm^2^ prod, 55°C and 0°C. (**D**) *In vivo* recordings of wide dynamic range (WDR) neurons in the deep dorsal horn (*n* = 45 littermate cells and *n* = 17 PV^DTA^ cells) demonstrate that PV^DTA^ cells show a 2-fold increase in action potentials fired to brush stimuli and an increase to cotton swab and vibration. They also demonstrate hyperexcitability to noxious mechanical and thermal stimulation. Data are mean ± SEM. Statistical analysis was performed using the multiple unpaired Student’s *t*-test. *P < 0.05; **P < 0.01; ***P < 0.001; ****P < 0.0001.

PV-Cre mice were crossed with Advillin-loxP-tdTomato-Stop-loxP-DTA mice to produce offspring with Cre-mediated DTA-ablation of the peripheral PV neurons (PV^DTA^) (Fig. 1A).^7,10^ A significant reduction of both PV mRNA (Fig. 1B) and protein was observed at the level of the soma (Fig. 1C and D), where 21.80 ± 1.65% of littermate neurons were PV-positive (PV+) compared to 6.91 ± 1.31% of PV^DTA^ DRG neurons (*P* < 0.001). In contrast, no significant changes in the number of PV+ neurons in the dorsal horn were observed (Supplementary Fig. 1). By crossing mice expressing a tdTomato reporter driven by the *Pvalb* promoter with Pirt-GCaMP6 mice, we obtained progeny where we could visualize the activity of tomato-labelled PV+ neurons and GCaMP6-expressing PV− neurons using *in vivo* calcium imaging (Supplementary Fig. 2). Our data suggest that <10% of recorded neurons in L4 DRG are PV+ as previously indicated^13^ and these neurons respond to vibration, as well as noxious mechanical and noxious heat stimuli (Supplementary Fig. 2). We next used *in vivo* GCaMP imaging to examine changes in neuronal excitability in PV^DTA^ mice at the level of the DRG. Calcium transients in sensory neurons of PV^DTA^; GCaMP3 animals were compared with control PV; GCaMP3 mice or DTA; GCaMP3 mice (Fig. 2A). The peak fluorescence of PV^DTA^ neurons was significantly increased in response to 150 g/cm^2^ noxious mechanical prod stimulation (2.71 ± 0.27 ΔF/F0 in PV^DTA^ mice compared to 1.05 ± 0.11 ΔF/F0 in littermate mice; *P* < 0.0001) (Fig. 2B). We also observed enhanced calcium flux in response to 55°C noxious heat (2.57 ± 0.22 ΔF/F0 in PV^DTA^ mice compared to 1.41 ± 0.10 ΔF/F0 in littermate mice; *P* < 0.001) and to 0°C noxious cold stimulation (3.46 ± 0.45 ΔF/F0 in PV^DTA^ mice compared to 0.92 ± 0.17 ΔF/F0 in littermate mice; *P* < 0.001) (Fig. 2C).

We further examined the consequences of peripheral PV+ neuron ablation on neural coding in the dorsal horn *in vivo*. We recorded extracellular spikes (action potentials) from WDR neurons located in the deep dorsal horn (laminae III–V), a region of the spinal cord that receives input from both mechano- and thermo-sensitive innocuous and noxious afferents. In PV^DTA^ animals we observed enhanced action potential firing in response to innocuous dynamic mechanical stimulation with brush stimulation (423.1 ± 98.1 in PV^DTA^ mice compared to 202.2 ± 34.3 in littermate mice; *P* < 0.01), cotton swab stimulation (530.1 ± 118.9 in PV^DTA^ mice compared to 309.1 ± 40.9 in littermate mice; *P* < 0.05) and vibratory tuning fork (521.1 ± 118.6 in PV^DTA^ mice compared to 320.8 ± 34.1 in littermate mice; *P* < 0.05) (Fig. 2D). Moreover, WDR neurons in PV^DTA^ mice were significantly more excitable in response to 55°C noxious heat (683.2 ± 176.2 in PV^DTA^ mice compared to 277.2 ± 25.5 in littermate mice; *P* < 0.001) and 0°C noxious cold stimulation (274.5 ± 77.88 in PV^DTA^ mice compared to 120.6 ± 15.6 in littermate mice; *P* < 0.01) (Fig. 2D). Aligned with our observation of enhanced evoked calcium flux at the level of the DRG, dorsal horn cells in PV^DTA^ mice also showed augmented action potential firing to noxious mechanical stimulation with prod (150 g/cm^2^) (794.7 ± 200.8 in PV^DTA^ mice compared to 499.2 ± 50.04 in littermate mice; *P* < 0.05) and with pinch (763.1 ± 213.2 in PV^DTA^ mice compared to 364.8 ± 36.07 in littermate mice; *P* < 0.01). We also examined the effect of PGE_2_ on GCaMP activity in DRG and spinal cord WDR electrophysiological activity in PV^DTA^ and littermate mice; administration of PGE_2_ generally sensitized evoked activity of DRG and dorsal horn neurons by noxious stimuli in littermate mice, but did not alter evoked DRG calcium transients or dorsal horn action potential firing in PV^DTA^ mice (Supplementary Fig. 3). However, we did observe enhanced spontaneous action potential firing of dorsal horn neurons in PV^DTA^ mice following PGE_2_ (9660 ± 3217 in PV^DTA^ mice before PGE_2_ compared to 13979 ± 4817 after PGE_2_; *P* < 0.05) in contrast to insignificant change in ongoing activity of neurons in littermate controls (Supplementary Fig. 3).

### Increased mechanical sensitivity and spontaneous pain in PV^DTA^ mice

**Figure 3:**
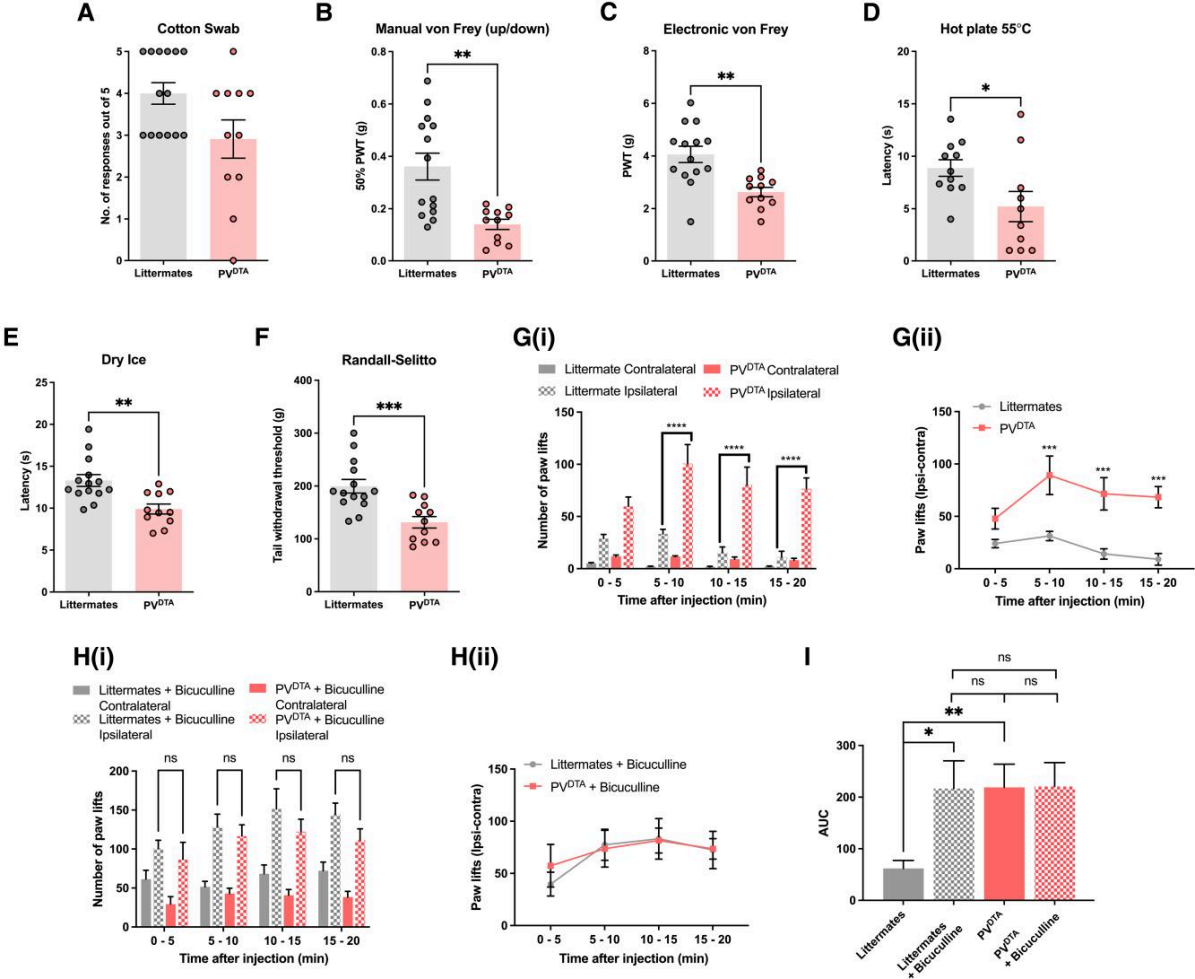
**Acute thermal and mechanical pain thresholds are reduced and spontaneous inflammatory pain behaviour augmented in PVDTA mice**. (**A**) Cotton swab test shows small differences between PVDTA mice and controls (*P* = 0.08, Mann-Whitney test). Mechanical pain thresholds are lower in PVDTA mice assessed using (**B**) manual up/down von Frey and (**C**) electronic von Frey assays, as are thermal pain thresholds using the (**D**) hot plate and (**E**) dry ice assays. (**F**) The Randall-Selitto assay demonstrates noxious mechanical hyperalgesia in PVDTA animals. Data are mean ± SEM. using unpaired Student’s *t*-test (littermate *n* = 11–14; PVDTA *n* = 10–11). [**G**(**i)**] PVDTA mice demonstrate enhanced spontaneous pain measured with hind paw lifts following intraplantar (i.pl.) administration of 20 µl 500 mM PGE_2_. [**G**(**ii**)] Paw lifts are the mean number of contralateral paw lifts subtracted from ipsilateral paw lifts in animals administered i.pl. PGE_2_. [**H**(**i**)] The experiments described in **G**(**i**) were repeated with 200 µM Bicuculline co-administered with i.pl. PGE_2_. Littermates also demonstrate enhanced spontaneous pain, comparable to PVDTA animals. [**H**(**ii**)] The mean number of contralateral paw lifts subtracted from ipsilateral paw lifts in animals co-administered i.pl. Bicuculline and i.pl. PGE_2_. Data are mean ± SEM. Statistical analysis was performed using repeated measures two-way ANOVA with Tukey’s multiple comparisons test. (**I**) The area under curve analyses of graphs **G**(**ii**) and **H**(**ii**) demonstrating enhanced response of littermates to i.pl. PGE_2_ in combination with bicuculline, comparable to the enhanced behavioural responses of PVDTA mice injected with PGE2 alone. Data are mean ± SEM. using unpaired Student’s *t*-test (littermates *n* = 7; PVDTA *n* = 6). *P < 0.05; **P < 0.01; ***P < 0.001; ****P < 0.0001.

In behaving mice, we observed that ablating peripheral PV+ neurons causes significant deficits in motor coordination^14^ (Supplementary Fig. 4). However, we were still able to measure withdrawal responses to noxious stimuli and quantitate pain thresholds in the PV^DTA^ mice^15^ and overall observed behavioural hypersensitivity of PV^DTA^ animals to noxious mechanical and thermal stimuli, consistent with the calcium imaging and electrophysiological findings. Behavioural responses to dynamic innocuous mechanical stimulation (Fig. 3A) were normal (2.91 ± 0.46 in PV^DTA^ mice compared to 4.00 ± 0.26 in littermate mice; *P* > 0.5). In contrast, we observed a robust mechanical hypersensitivity in PV^DTA^ animals in the manual (Fig. 3B; 0.14 ± 0.02 g in PV^DTA^ mice compared to 0.36 ± 0.05 g in littermate mice; *P* < 0.01), and electronic (Fig. 3C; 2.63 ± 0.18 g in PV^DTA^ mice compared to 4.06 ± 0.31 g in littermate mice; *P* < 0.01) von Frey assays, as well as the Randall-Sellitto test for noxious mechanical sensation (Fig. 3F; 131.3 ± 10.89 g in PV^DTA^ mice compared to 199.5 ± 12.88 g in littermate mice; *P* < 0.01). Sensitivity to noxious heat (5.21 ± 1.44 s in PV^DTA^ mice compared to 8.88 ± 0.79 s in littermate mice; *P* < 0.05) and cold (9.90 ± 0.60 s in PV^DTA^ mice compared to 13.30 ± 0.70 s in littermate mice; *P* < 0.05) was also enhanced in PV^DTA^ mice (Fig. 3D and E).

Following intraplantar administration of the inflammatory mediator PGE_2_, we recorded spontaneous nocifensive behaviours that were markedly enhanced in the PV^DTA^ mice up to 3-fold (100.7 ± 18.2 over 5 min) compared to littermate animals (33.3 ± 4.4 over 5 min) (*P* < 0.0001) [Fig. 3G(i and ii)]. To test the hypothesis that PV+ DRG neurons are contributing to an inhibitory primary afferent drive on spinal cord excitability, we tested the effects of intraplantar bicuculline (200 µM in 20 µl) on PGE_2_ evoked spontaneous pain behaviour [Fig. 3H(i and ii)]. Intraplantar bicuculline significantly increased PGE_2_-driven spontaneous pain behaviour in littermate mice, but this effect is lost in the absence of PV neurons in DTA mice (Fig. 3I).

## Discussion

We used diphtheria toxin-mediated ablation of PV-expressing neurons to investigate the role of this subset of DRG cells in pain behaviour and neuronal excitability at the level of the DRG soma and spinal cord dorsal horn. Our data demonstrate that: (i) PV+ neurons are activated by vibratory, noxious mechanical and noxious thermal stimuli; (ii) ablation of PV+ neurons from the peripheral nervous system lowers withdrawal thresholds to noxious mechanical and thermal stimuli in awake behaving mice; and (iii) the expression of PV+ neurons in DRG is essential for neuronal responses to mechanical and thermal stimuli at the level of the soma and dorsal horn, as well as for augmented neuronal responses following acute inflammation with PGE_2_. Our data suggest that peripheral PV+ neurons are responsible for tonic inhibition of nociceptors at the level of the soma.

A comparison of modality-specific GCaMP signals in PV+ neurons compared to other DRG neurons showed that subsets of PV+ neurons respond to vibration, noxious mechanical and noxious heat stimuli (Supplementary Fig. 2). Mice lacking sensory input from PV+ neurons showed augmented action potential firing of dorsal horn neurons to vibration, noxious mechanical, as well as noxious heat and cold stimuli. These findings of enhanced neuronal excitability are in line with the behavioural hypersensitivity of PV^DTA^ animals to mechanical and thermal stimuli. Moreover, mice lacking sensory input from PV+ neurons show enhanced spontaneous pain behaviour and spontaneous actional potential firing of dorsal horn WDR neurons following an acute inflammatory insult with intraplantar PGE_2_. These findings are in line with the conventional gate control theory describing presynaptic control of synaptic transmission from large and small sensory afferents to ‘gate’ incoming information depending on the balance between these inputs.^3^ However, we also observed enhanced calcium flux at the DRG level in mice lacking sensory input from PV+ neurons compared to littermate controls, demonstrating the existence of a peripheral gate.

Enhanced nociceptor activity within the somata of sensory neurons in PV^DTA^ mice is consistent with a regulatory role of GABA within sensory ganglia. Sensory neurons have the synthetic enzymes and uptake transporters for GABA metabolism, and depolarization of sensory neurons results in GABA release, e.g. both genomic and protein expression of GAD67 and VGAT in mouse DRG.^6^ Interestingly, within the CNS of rats, individual habenula neurons are known to be both glutamatergic and GABAergic.^16^ Our data suggest that the same phenomenon occurs in sensory neurons. All sensory neuron subsets express VGLUT1, necessary for glutamatergic vesicle loading.^17^ The set associated with highest levels of parvalbumin, PSNF2, have lower levels of VGLUT2 mRNA than other sensory neuron subsets (Supplementary Table 2 compiled using the nomenclature produced by the Linnarsson group^13^). GABA uptake transporters are present in PV-expressing sensory neurons; we found that PSNF2 neurons that express high levels of PV mRNA also express the GABA uptake transporter SLC6A13 and the microtubule associated protein GPHN that clusters GABA_A_ receptors. PSNF3 neurons express both low levels of PV and GAD1 the glutamate decarboxylase enzyme that generates GABA from glutamate. Glycine transporters and glycine receptor GLRA1 are present at low levels in sensory neurons. GABA ionotrophic receptor GABRA1 is expressed at high levels by nociceptors and other sensory neurons. Recent work demonstrates that that focal administration of bicuculline can increase the firing rate measured in the mouse dorsal root^18^—a finding that is consistent with another observation that application of bicuculline through a L5-DRG-implanted cannula induces nocifensive behaviour.^6^ Our observation that GABA receptor antagonism with intraplantar administration of bicuculline increases nocifensive responses in littermates, but not PV^DTA^ mice, supports our findings that PV+ peripheral sensory neurons exert a tonic inhibitory tone on second order neurons in the dorsal horn. It is interesting that WDR activity in response to innocuous stimuli is also enhanced in the PV^DTA^ mice, suggesting that tonic GABA release from PV neurons may act on the somata of all classes of sensory neurons within the ganglia.

Despite the genetically defined specificity of DTA ablation, one potential confounding factor in our study is that the toxin is expressed *in utero*, such that neuronal function is compromised from birth. However, this loss-of-function approach is a successful tool that has revealed crucial aspects of sensory circuitry and of homeostatic functions within the nervous system in previous studies.^19–21^ Another confounding factor is the theoretical framework of membrane depolarization mediated by GABA receptors through quantum tunnelling; however, the work in our study was not focused on membrane excitability of individual cells, but rather *in vivo* outcomes that capture the polysynaptic constitution of pain behaviour.^22^ Importantly, our data demonstrate that the robust effect of GABA receptor antagonism on enhanced nocifensive responses in littermate mice following PGE_2_ administration is lost in the absence of PV neurons in DTA mice (Fig. 3I). Therefore, our findings suggest that the enhanced excitatory drive observed behaviourally in PV^DTA^ mice is largely dependent on peripheral GABA circuitry.

In summary, we observed that deletion of PV+ sensory neurons leads to both enhanced pain behaviour and increased excitation of somata within DRG. Our findings demonstrate that PV+ neurons mediate tonic inhibition of nociceptive activity in peripheral sensory neurons within DRG. Whether these same, and/or additional neurons also control sensory input within the dorsal horn remains to be established. Recent studies of the analgesic effects of exogenous application of GABA to sensory ganglia are consistent with a substantial contribution to gate control within sensory ganglia.^6^ These findings extend the model of Melzack and Wall,^2^ and present a therapeutic opportunity for manipulating the peripheral gate outside the CNS.

## Supplementary Material

awad182_Supplementary_Data

## Data Availability

Data are available at Figshare: http://dx.doi.org/https://doi.org/10.6084/m9.figshare.20431200.v1; http://dx.doi.org/https://doi.org/10.6084/m9.figshare.22961822.v2; http://dx.doi.org/https://doi.org/10.6084/m9.figshare.20431179.v1; http://dx.doi.org/https://doi.org/10.6084/m9.figshare.20399205.v1; http://dx.doi.org/https://doi.org/10.6084/m9.figshare.20431170.v1; http://dx.doi.org/https://doi.org/10.6084/m9.figshare.20399190.v1; http://dx.doi.org/https://doi.org/10.6084/m9.figshare.20399160.v1.
