# Supplementary material for: Gate control of sensory neurotransmission in peripheral ganglia by proprioceptive sensory neurons
Source: Brain. 2023 May 30;146(10):4033–9. doi: 10.1093/brain/awad182 (PMC10549771; doi:10.1093/brain/awad182)
Supplement: awad182_Supplementary_Data [file awad182_supplementary_data.pdf]

<u>Supplementary Materials for:</u>

Gate Control of Sensory Neurotransmission in Peripheral Ganglia by Proprioceptive Sensory Neurons

Alice M. Fuller[1,2], Ana Luiz[2], Naxi Tian[2], Manuel Arcangeletti[2], Federico Iseppon[2], Jane E. Sexton[2], Queensta Millet[2], Sara Caxaria[1], Niloofar Ketabi[1], Petek Celik[1], John N. Wood[2*], Shafaq Sikandar[1*]

[1] William Harvey Research Institute, Charterhouse Square, Queen Mary University of London; London, EC1M 6BQ, UK.

[2] Wolfson Institute for Biomedical Research, University College London, Gower Street, London WC1E 6BT, UK.

*Corresponding authors: s.sikandar@qmul.ac.uk, j.wood@ucl.ac.uk

This file includes:

Materials and Methods, with additional references
Figs. S1 to S4
Table S1 and S2

Materials and Methods

Immunohistochemistry (spinal cord)

Littermate and PV^{DTA} mice were transcardially perfused with 4% paraformaldehyde. Isolated lumbar spinal cord were post-fixed at 4°C for 2h, followed by 30% w/v sucrose in 1XPBS at 4ºC for 48h before being mounted in OCT medium (Tissue-Tek) and stored at -80°C. Free-floating 30µm sections were washed in PBST followed by incubation with blocking buffer (3%NGS) for 60min at RT. Tissue was then incubated with anti-Parvalbumin antibody (Abcam, ab11427) diluted in TTBS (1:5000), at RT overnight. Tissue was incubated with an AlexaFluor594 goat anti-rabbit secondary antibody (Thermofisher A11037) diluted in TTBS (1:500) for 2hr at RT. Images were captured from ProLong ™ Gold mounted slides using a NanoZoomer S60 Digital slide scanner (Hamamatsu) and the Texas Red filter. ImageJ was used for cell counts.

<u>Behavioural studies</u>

Motor co-ordination tests

Beam test:

Mice were placed at one end of a beam of ~1 cm^2 cross sectional surface area, which was above a box containing soft bedding. A white light, which was designed to deter the mice, was placed at the start of the beam walk, while food was placed at the end of the beam to attract them. After at least 3 training attempts, mice were filmed walking along the beam from one side to the other. Littermate mice are able to use their tail for balance and grip the beam without issue. Mice with

motor and coordination deficits are typically unable to grasp or balance on the beam and can fall from it.

Set speed rotarod:

The rotarod (IITC Life Science) apparatus was set such that littermate mice were able to stay on the rod for the entire duration of the test. The rod rotated at 5 rpm for a period of 60 seconds. The latency of the animal to fall from the rod was recorded; if mice held onto the rod for 3 consecutive revolutions this also counted as a fall. The test was repeated 3 times per mouse and an average taken.

Activity test:

Mice were placed in an open field enclosure and video recording software was used to assess activity and rearing of mice when in a novel environment for a period of 5 minutes. The total distance travelled as well as the number of rearings were monitored using automated software for this period.

Grip force:

The automated Grip-Strength Meter (Ugo Basile) was used to assess the grip strength and duration of grip in mice. Mice were placed on a plastic grate with all four paws in contact with the grate. They were then gently pulled by the tail until they released the grate from their grip.

The duration mice held onto the grate and the maximum force which was applied to the grate was recorded. The test was repeated 3 times per mouse and an average taken.

Reaching score:

Each animal was held in the air by the tail, 5 cm away from a horizontal surface for 5 seconds. A wild type mouse should demonstrate a reaching movement towards the horizontal surface, whereas animals with proprioceptive deficits are inclined to curl towards the abdomen. A scoring system was used to quantify this; if the mouse reached its forelimbs towards the surface, it received a score of 1. If the mouse curled towards the abdomen, it received a score of 0. A partial curl received a score of 0.5.

Mechanical sensitivity tests

Cotton swab test:

The cotton swab test was used as a measure of innocuous dynamic sensitivity.[1] Mice were habituated in modular enclosures placed on a mesh platform for a minimum of 1 hr prior to testing. A cotton swab was "puffed out" so that the head was approximately 3 times the normal size. This was stroked across the plantar surface of the paw in a ~1 second sweep 5 times with a minimum of a minute between each stroke. A response was counted if either a withdrawal from the cotton swab was observed or a flutter or shake of the paw.

Up-down von Frey:

Tactile punctate sensitivity was assessed using von Frey hairs (Bioseb) applied to the plantar surface of the paw using the up-down method for obtaining the 50% paw withdrawal threshold.[2] Animals were habituated to von Frey chambers (modular enclosures placed on a mesh platform) for a minimum of 1 hr before the experiment. Von Frey filaments where then applied

perpendicularly to the paw, until buckling, always beginning with the filament that provides 0.4 g of force. If a response (i.e. a lift, flick, or shake of the paw) was observed during or immediately after filament application, the next filament down (in this case 0.16 g) was applied in the following round of testing, and if there was no response, the next filament up (0.6 g) was applied and so on until a minimum of 6 recordings had been made.

Dynamic plantar aesthesiometer (electronic von Frey):

As another means of assessing mechanical threshold, a dynamic plantar aesthesiometer was used (Ugo Basile). In this instance a single unbending filament was applied to the hind paw with increasing force until the paw was withdrawn. A major benefit to using the electronic von Frey apparatus over manual von Frey filaments is that this method produces less variability between datasets. It should be noted that the electronic von Frey assay produces paw withdrawal thresholds that are much higher than with manual von filaments, a typical threshold for C57BL/6 mice being ~5-6g.[3] Mice were habituated in modular enclosures placed on a mesh platform for a minimum of 1 hr prior to testing. The touch stimulator unit was positioned under the target area of the paw and the protocol initiated. This drove a movable force actuator (a metal 0.5 mm filament) set to apply a maximum weight of 10 g with a ramp rate of 20 s (ramp speed 0.5 g/s).[4] The force at which the animal withdrew its paw from the stimulus was automatically recorded by the machine. This was repeated on each hind paw 3 times and an average taken.

Randall-Selitto:

Noxious mechanical sensitivity was assessed using the Randall-Selitto assay.[5] A bench-top, as opposed to a hand-held device, was used (Ugo Basile).Mice were restrained in a clear plastic tube and acclimatised for 5 minutes. A blunt force was then applied to the tail with increasing pressure until a pain response was evoked.[6] A pain response was considered to be either a

vocalisation, or an escape reaction. The experiment was then stopped, and the force recorded. This was repeated on each animal 3 times and an average taken.

Thermal sensitivity tests

Hotplate:

The hot plate assay is a means of assessing heat sensitivity believed to be reliant on supraspinal processing.[7,8] A hot/cold plate (Ugo Basile) was used for this experiment. Mice were habituated to the apparatus at room temperature for 2 minutes the day before testing. On the day of testing, a mirror was placed behind the plate in order to observe the animal from all angles, and the plate was heated and held at 55 ± 0.2ºC. The mouse was placed on the plate and the timer started. The time at which the mouse exhibited a nocifensive behaviour (hind paw licking, shaking, or jumping etc.) was recorded and the mouse immediately removed from the plate.

Dry ice:

As first described by Brenner *et al.*[9] the dry ice assay was used to assess sensitivity to cooling of the hind paw. Mice were habituated on a glass base (6 mm thickness) in perspex modular enclosures for a minimum of 1 hr prior to testing. A pellet of compacted dry ice power was applied to the glass underneath the hind paw, which provides a ramping cooling stimulus, with temperature ranges between 5–12°C. The latency of the mouse to withdraw their hind paw from the stimulus was measured. This was repeated on each animal 3 times and an average taken.

PGE$_2$ and bicuculline administration and behavioural testing

Administration of prostaglandin E2 (PGE$_2$) is a model of acute inflammation, resulting in thermal and mechanical hypersensitivity in the region of administration.[10,11] Mice were habituated in transparent modular enclosures placed on a mesh platform for a minimum of 1 hr..

Each mouse then received a single subcutaneous injection of 20 µl 500 mM PGE_2 (Sigma) in 1% ethanol and saline into the ventral aspect of the ipsilateral hind paw. Mice were placed immediately back into their modular enclosures and their activity video recorded for 20 minutes. Activity recordings were analysed after the assay for incidences of paw lifts as an indicator of non-stimulus evoked nocifensive responses. In the case of the PGE_2 + bicuculline experiments, a combined i.pl. injection of 500 mM PGE_2, and 200 µM bicuculline in a total volume of 20 µl saline was administered.

In vivo GCaMP imaging and analysis

Once the L4 DRG was exposed, the mouse was clamped in position at the vertebral column rostral to the laminectomy using custom made clamps. The ipsilateral hind paw was secured to allow for application of stimuli. The stimulation protocol is described in Table S1. All *in vivo* data was acquired using LASX software (Leica). Videos were converted into TIFF format and analysed using ImageJ software (NIH). The TIFF videos were first stabilised for XY movement using the Turboreg ImageJ plugin, and ROIs were manually drawn within the cytoplasm of identified neurons. Raw data was extracted in the form of averaged pixels per ROI per frame and analysed in Microsoft Excel. To determine whether a neuron was responsive to a given stimulus, the raw traces were first smoothed by averaging the preceding four frames of any test frame to reduce noise. Then the derivative of each frame was taken as $\Delta F/\Delta t$ (change from one frame to the next), where F is fluorescence and t is time. A neuron was included as a responder to a given stimulus if the following was true: $\Delta Fstim/\Delta t > [(\Delta Fbasal/\Delta t)+4sbasal]$, where Fstim is the maximum derivative value within a given stimulus application window. Fbasal is the average derivative baseline value (average of four frames preceding stimulus), and sbasal is the SD of the baseline derivative values. Peak fluorescence of a given neuron was determined by calculating

ΔF/F0 in smoothed traces, where ΔF/F0 =(Ft-F0)/F0. Ft is the fluorescence at time t, and F0 the minimum fluorescence over the entire baseline and stimulation period.[12]

In vivo electrophysiology recording

Once an appropriate neuron was isolated a 5 minute recording was taken prior to stimulation to record baseline spontaneous activity. To assess evoked activity the same stimuli used for the *in vivo* imaging experiments were applied to the ipsilateral glabrous skin of the hind paw (Table S1). This stimulation protocol also included 8 g and 26 g von Frey filaments. Ethyl chloride was applied for a duration of 1 second as a further measure of noxious cooling, and the evoked activity measured over a duration of 10 s. This particular cooling assay cools skin to noxious temperatures of 5°C or below.[13] Spontaneous activity to ethyl chloride was also recorded for a duration of 60 s after the initial 10 s recording. Unless otherwise stated, all other stimuli were applied to the hind paw for a duration of 10 s. Once this protocol was completed, the animals were injected in the same hind paw with 20 µl of 500 µM PGE_2, and another 5 minute recording was made to assess the extent of non-stimulus evoked activity. The stimulation protocol was then repeated. During data analysis, neurons were discarded if they did not produce a minimum of 100 spikes to a brush stimulus, a minimum of 50 spikes for an 8 g von Frey stimulus, and a response to noxious heat. To count as a response, a minimum of 10% change from baseline was required.[14]

Statistical analysis

Statistical analysis was performed where appropriate using GraphPad Prism 9 Software. The mean ± SEM was calculated for all data where applicable. To compare groups Student's unpaired t tests, Student's paired t tests, regular two-way ANOVA with Tukey's multiple comparison test, and regular one-way ANOVA with Dunnet's multiple comparison test, were performed. Mann-Whitney test was used for comparisons within discrete datasets. Data are presented as Mean ± SEM. *$p<0.05$; **$p<0.01$; ***$p<0.001$; ****$p<0.0001$.

Results

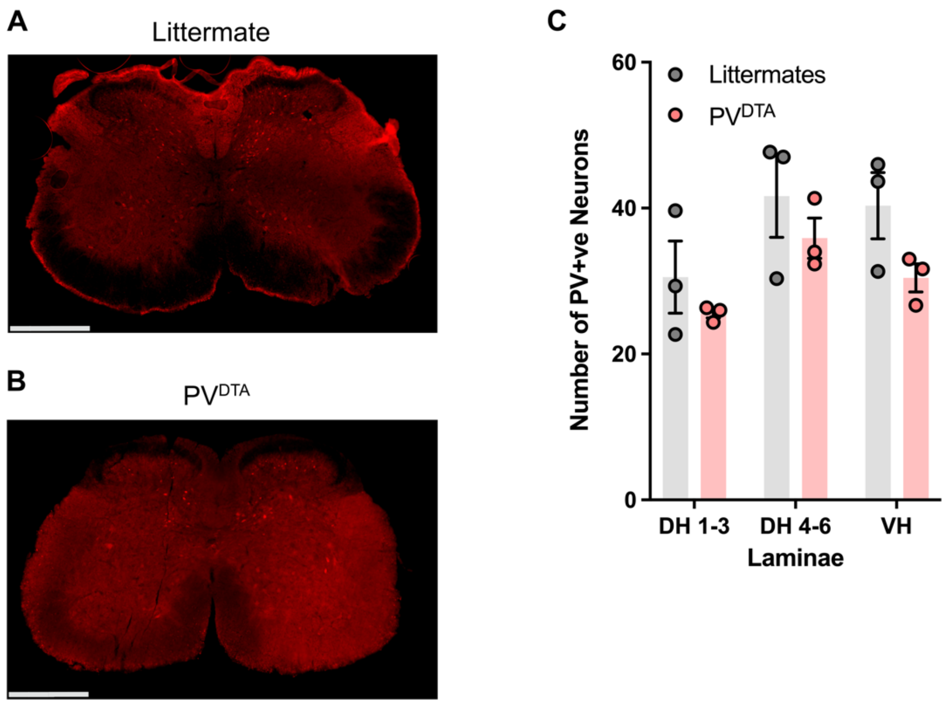

Fig. S1. DTA ablation of PV+ DRG neurons does not alter the number of PV+ neurons in the spinal cord. (A) Representative image of a lumbar spinal cord section from an adult littermate control versus **(B)** a PV^{DTA} mouse, after immunofluorescence staining for parvalbumin protein. **(C)** Quantification of PV+ neurons in lamina 1-6 of the dorsal horn (DH), and the ventral horn (VH) (*n* =3 per genotype) Data are shown as mean ± SEM with unpaired Student's t test. The scale bars represent 500µm.

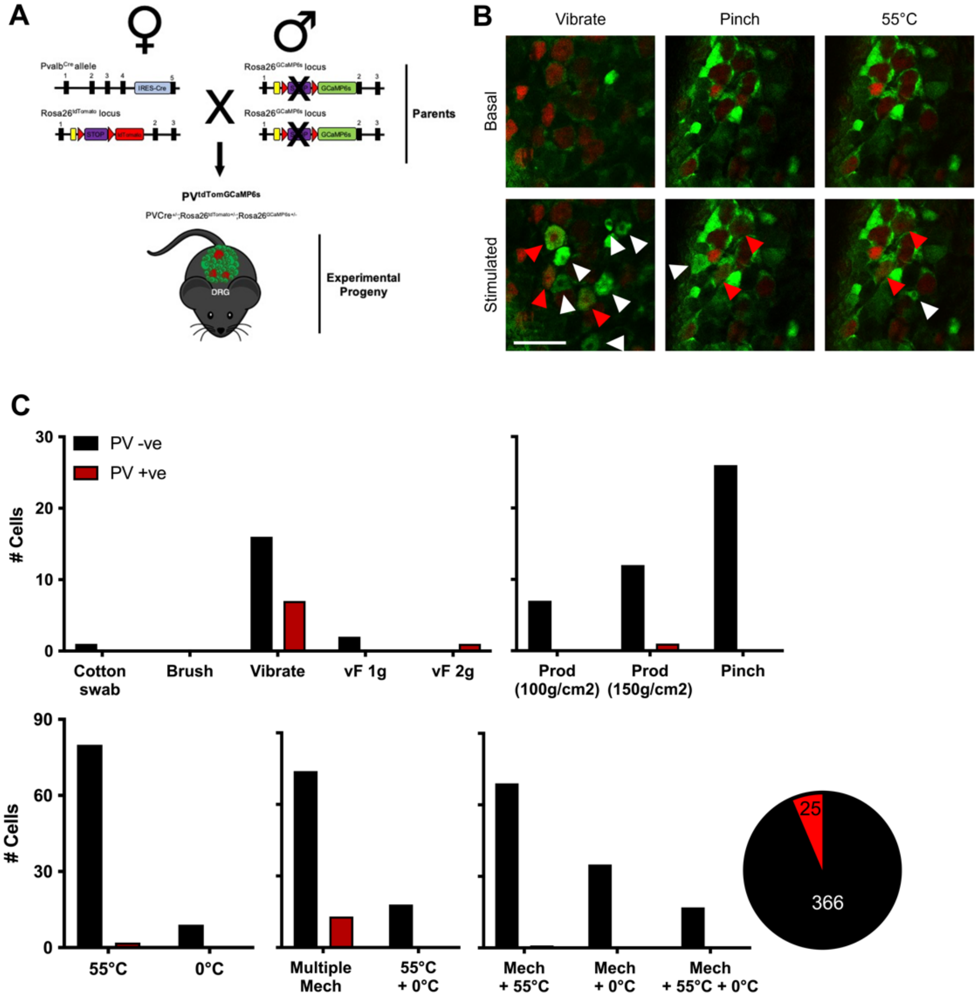

Fig. S2. Mechanical and thermal coding of PV and non-PV neurons in L4 DRG quantified with *in vivo* calcium imaging. (A) The red arrowheads denote loxP sites. A cross over the Stop codon of the Rosa26GCaMP6s locus indicates it's deletion and subsequent constituent global expression of GCaMP6s. Female mice heterozygous for the Pvalb-Cre allele and for the Rosa26-LSL-tdTomato allele were bred to male mice homozygous for global GCaMP6s, to produce experimental progeny that were heterozygous for Pvalb-Cre;Rosa26-LSL-tdTomato;GCaMP6s (PVTom;GCaMP6s mice). The DRG neurons of these mice expressed the red fluorescent reporter tdTomato in PV+ neurons on a background of green GCaMP6s expression. **(B)** Representative images of parvalbumin expressing neurons (red) in mice expressing GCaMP6s in all sensory neurons (green) *in vivo*. Red arrows indicate PV+ cells responding to mechanical or

thermal stimulation of the hind paw receptive field, white arrows indicate PV- cells. **(C)** Total number of PV+ and PV- DRG neurons responding to mechanical and thermal stimulation of the hind paw receptive field. Total number of responding cells represented by the pie chart.

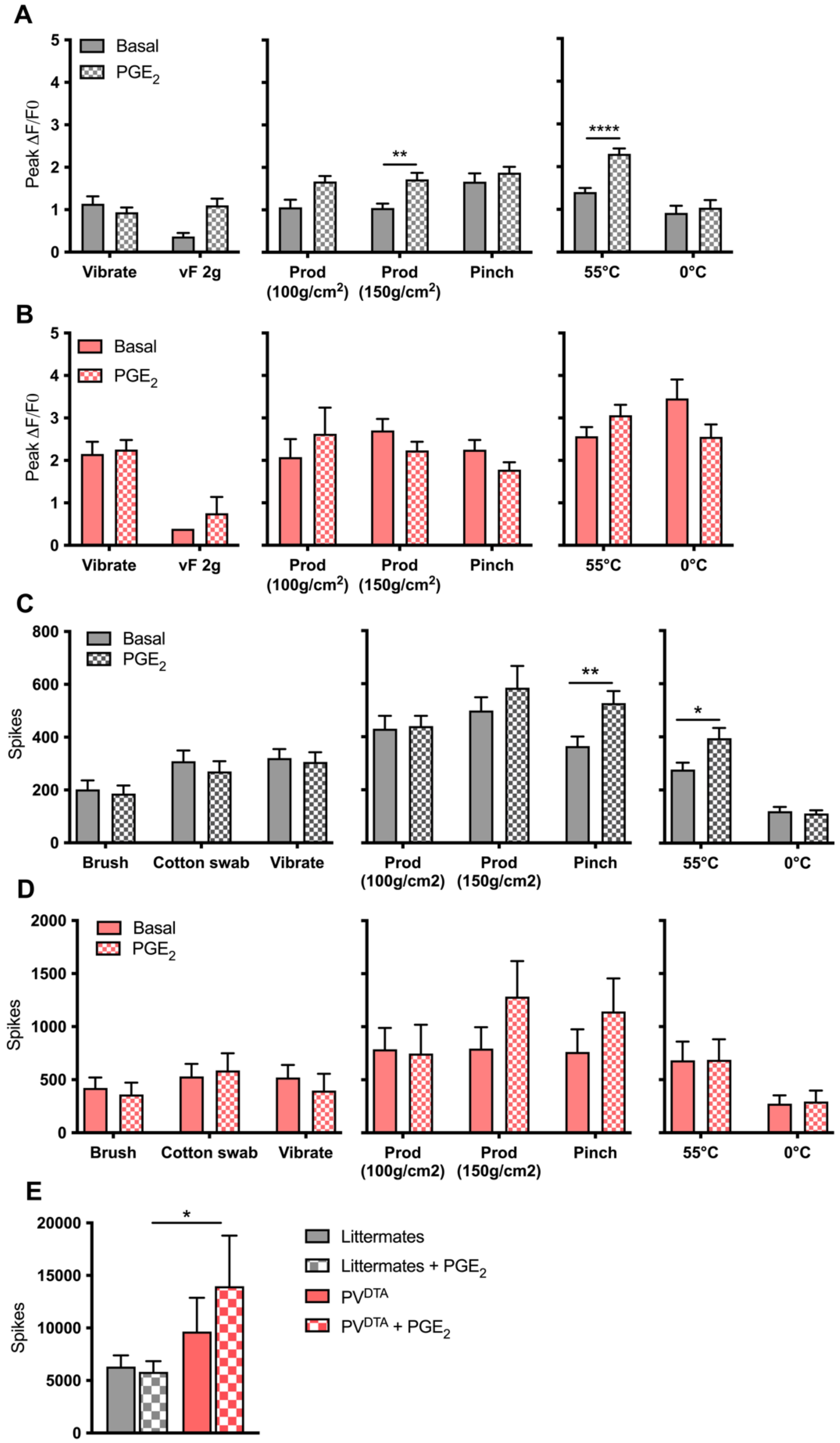

A
Basal
PGE2
Peak ΔF/F0
**
****
Vibrate
vF 2g
Prod (100g/cm2)
Prod (150g/cm2)
Pinch
55°C
0°C
B
Basal
PGE2
Peak ΔF/F0
Vibrate
vF 2g
Prod (100g/cm2)
Prod (150g/cm2)
Pinch
55°C
0°C
C
Basal
PGE2
Spikes
**
*
Brush
Cotton swab
Vibrate
Prod (100g/cm2)
Prod (150g/cm2)
Pinch
55°C
0°C
D
Basal
PGE2
Spikes
Brush
Cotton swab
Vibrate
Prod (100g/cm2)
Prod (150g/cm2)
Pinch
55°C
0°C
E
Spikes
*
Littermates
Littermates + PGE2
PVDTA
PVDTA + PGE2

Fig. S3. PGE_2 enhances DRG GCaMP activity and WDR neuronal activity in response to noxious peripheral stimulation in littermate mice, but has no effect in PV^{DTA} mice

Peak fluorescence (activity) of neurons in littermate (grey) **(A)** and PV^{DTA} (pink) **(B)** animals before and after intraplantar PGE_2 injection. Activity of neurons responding to 150g/cm^2 prod and 55°C is significantly increased after PGE_2 injection in the littermate. There is no significant change in activity as a consequence of PGE_2 injection in PV^{DTA} animals. **(C)** An increase in action potential firing is observed in the WDR neurons of the DH in littermate animals in response to noxious mechanical pinch, and to 55ºC, in the presence of PGE_2. **(D)** PV^{DTA} cells are not sensitized to PGE_2 administration. **(E)** Stimulus independent (spontaneous) activity of WDR neurons recorded in basal conditions (5 minutes prior to application of stimuli) and following i.pl. PGE_2 (5 minutes after administration) is doubled in PV^{DTA} cells. Data are mean ± SEM. Statistical analysis was performed using the multiple unpaired Student's t test. (*n*=4 mice per group). *$p<0.05$; **$p<0.01$; ***$p<0.001$; ****$p<0.0001$.

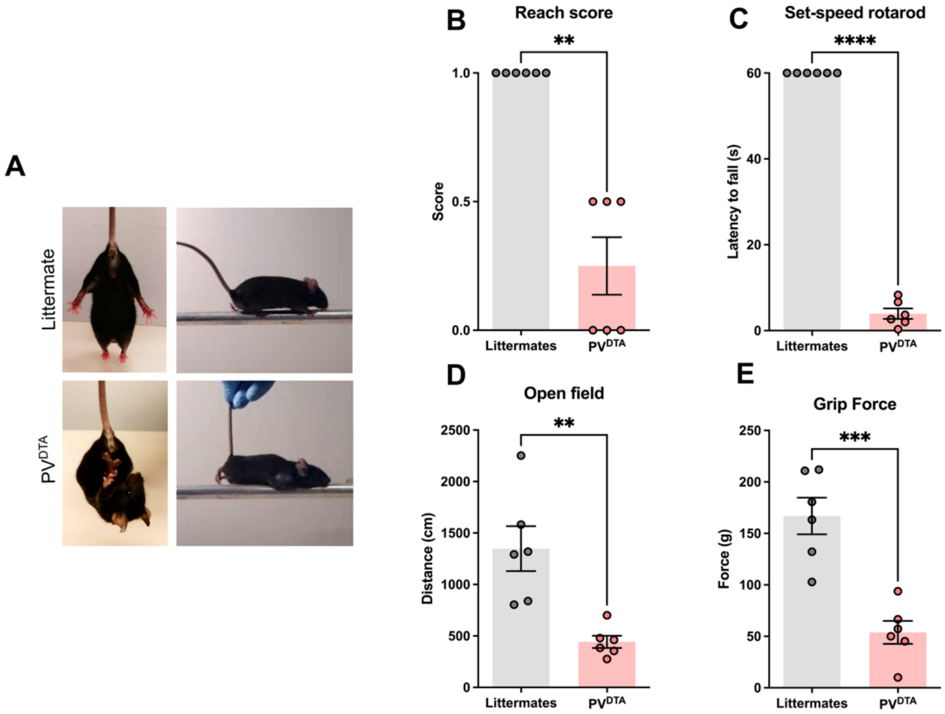

Fig. S4. PV^{DTA} mice have significant deficits in motor function and coordination.

(A) Representative images of age-matched adult PV^{DTA} mouse versus littermate control showing reach and beam tests. **(B)** Reach score assigned according to ability of mouse to reach towards the ground while held by the tail. Data displayed as mean ± SEM with Mann-Whitney test. **(C)** Time spent on rotarod apparatus set at a constant speed of 5 rpm with a maximum test time of 60 s. **(D)** Distance travelled in 5 minutes in an open field chamber. **(E)** Total force exerted by all four limbs on grip force grid attachment. (*n* =6 per group) Data are shown as mean ± SEM with unpaired Student’s t test unless specified otherwise.

Time	Stimulus		Duration
00:30	Mechanical	Cotton Swab	10 s
01:00		Brush	
01:30		Vibrate (128 Hz)	
02:00		von Frey 1.0 g	10 Applications
02:30		von Frey 2.0 g	
03:00		Prod 100 g/cm2	10 s
03:30		Prod 150 g/cm2	
04:00		Pinch	
04:30	Thermal	55 °C Water	10 s
05:00		0 °C Water	
Intraplantar injection of 20 µl 500 µM PGE2, wait 5 minutes then repeat protocol			

Table S1. ***In vivo*** **GCaMP imaging stimulation protocol**

The stimulation protocol began with the most innocuous stimuli and gradually increased in intensity, ending in the most noxious in order to limit sensitisation throughout the duration of the protocol (the gradual increase in intensity is depicted as the transition from green to red). The protocol was designed to encompass as many mechanical modalities as possible, including dynamic stimuli such as cotton swab and bush, which were swept over the hind paw for a duration of 10 s. Unlike the other stimuli that maintained contact with the skin for 10 s, von Frey filaments were applied to the hind paw 10 times in 10 different regions to ensure as much of the paw was stimulated as possible, thus increasing the chance of visualising activated neurons at the DRG. Pinch (using curved forceps to avoid tissue damage), and 55°C and 0 °C water were considered noxious.

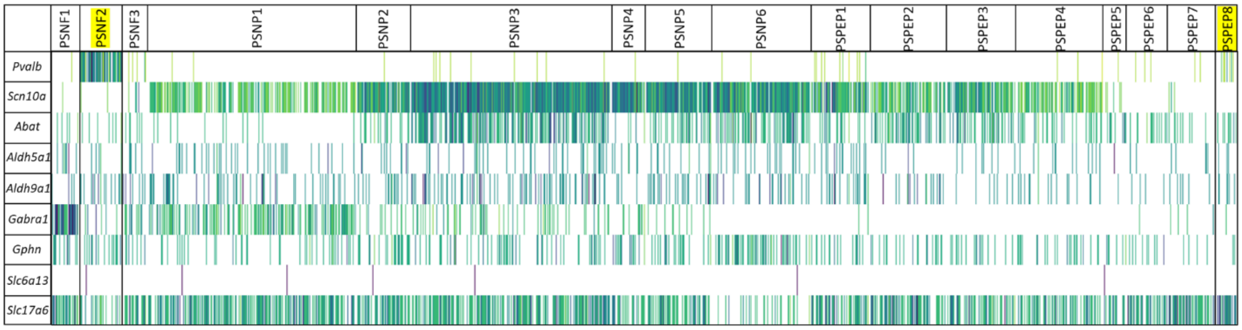

Table S2. Transcriptional profiles of GABA-related genes within two distinct PV+ populations of sensory neurons

Data extracted from the Linnarsson 'Peripheral sensory neurons' dataset and visualised using the Loom Viewer web tool (http://loom.linnarssonlab.org/). Genes are listed on the left-hand side, with *Pvalb*, and *Scn10a* placed at the top for reference, and GABA-related genes listed underneath. X axis labels refer to DRG clusters as defined by Usoskin et al. (2015) and Zeisel et al. (2018). The two PV-rich clusters PSPEP8 and PSNF2 are highlighted in yellow.

Pvalb encodes parvalbumin; *Scn10a,* Na_V1.8; *Abat,* ABAT; *Aldh5a1,* ALDH5A1; *Aldh9a1,* ALDH9A1; *Gabra1,* GABRA1; *Gphn*, gephyrin; *Slc6a13,* GAT2; *Slc17a6*, VGLUT 2.

References

1 Garrison, S. R., Dietrich, A. & Stucky, C. L. TRPC1 contributes to light-touch sensation and mechanical responses in low-threshold cutaneous sensory neurons. *J Neurophysiol* **107**, 913-922, doi:10.1152/jn.00658.2011 (2012).

2 Chaplan, S. R., Bach, F. W., Pogrel, J. W., Chung, J. M. & Yaksh, T. L. Quantitative assessment of tactile allodynia in the rat paw. *J Neurosci Methods* **53**, 55-63 (1994).

3 Deuis, J. R., Dvorakova, L. S. & Vetter, I. Methods Used to Evaluate Pain Behaviors in Rodents. *Front Mol Neurosci* **10**, 284, doi:10.3389/fnmol.2017.00284 (2017).

4 Emery, E. C., Young, G. T., Berrocoso, E. M., Chen, L. & McNaughton, P. A. HCN2 ion channels play a central role in inflammatory and neuropathic pain. *Science* **333**, 1462-1466, doi:10.1126/science.1206243 (2011).

5 Randall, L. O. & Selitto, J. J. A method for measurement of analgesic activity on inflamed tissue. *Arch Int Pharmacodyn Ther* **111**, 409-419 (1957).

6 Minett, M. S. *et al.* Endogenous opioids contribute to insensitivity to pain in humans and mice lacking sodium channel Nav1.7 *Nature Communications* (2015).

7 Giglio, C. A., Defino, H. L., da-Silva, C. A., de-Souza, A. S. & Del Bel, E. A. Behavioral and physiological methods for early quantitative assessment of spinal cord injury and prognosis in rats. *Braz J Med Biol Res* **39**, 1613-1623, doi:10.1590/s0100-879x2006001200013 (2006).

8 Woolfe, G. & Macdonald, A. D. The evaluation of the analgesic action of pethidine hydrochloride (demerol). *Journal of Pharmacology and Experimental Therapeutics* **80**, 300-307 (1944).

9 Brenner, D. S., Golden, J. P. & Gereau, R. W. t. A novel behavioral assay for measuring cold sensation in mice. *PLoS One* **7**, e39765, doi:10.1371/journal.pone.0039765 (2012).

10 Domenichiello, A. F., Wilhite, B. C., Keyes, G. S. & Ramsden, C. E. A dose response study of the effect of prostaglandin E2 on thermal nociceptive sensitivity. *Prostaglandins Leukot Essent Fatty Acids* **126**, 20-24, doi:10.1016/j.plefa.2017.08.015 (2017).

11 Kuhn, D. C. & Willis, A. L. Proceedings: Prostaglandin E2, inflammation and pain threshold in rat paws. *Br J Pharmacol* **49**, 183p-184p (1973).

12 Emery, E. C. *et al.* In vivo characterization of distinct modality-specific subsets of somatosensory neurons using GCaMP. *Sci Adv* **2**, e1600990, doi:10.1126/sciadv.1600990 (2016).

13 Leith, J. L., Koutsikou, S., Lumb, B. M. & Apps, R. Spinal Processing of Noxious and Innocuous Cold Information: Differential Modulation by the Periaqueductal Gray. *The Journal of Neuroscience* **30**, 4933-4942, doi:10.1523/jneurosci.0122-10.2010 (2010).

14 Sikandar, S. *et al.* Brain-derived neurotrophic factor derived from sensory neurons plays a critical role in chronic pain. *Brain* **141**, 1028-1039, doi:10.1093/brain/awy009 (2018).
